# Unveiling gender differences in psychophysiological dynamics: support for a two-dimensional autonomic space approach

**DOI:** 10.3389/fnhum.2024.1363891

**Published:** 2024-03-13

**Authors:** Yarden Menashri Sinai, Yaopeng X. J. Ma, Michal Abba Daleski, Sharon Gannot, Ronny P. Bartsch, Ilanit Gordon

**Affiliations:** ^1^The Gonda Multidisciplinary Brain Research Center, Bar Ilan University, Ramat-Gan, Israel; ^2^Department of Physics, Bar-Ilan University, Ramat-Gan, Israel; ^3^Department of Psychology, Bar-Ilan University, Ramat-Gan, Israel; ^4^The Faculty of Engineering, Bar Ilan University, Ramat-Gan, Israel

**Keywords:** cardiac autonomic balance (CAB), cardiac autonomic regulation (CAR), cross-system autonomic balance (CSAB), cross-system autonomic regulation (CSAR), respiratory sinus arrythmia (RSA), electrodermal activity (EDA), pre-ejection period (PEP), gender

## Abstract

**Introduction:**

To date, studies focusing on the connection between psychological functioning and autonomic nervous system (ANS) activity usually adopted the one-dimensional model of autonomic balance, according to which activation of one branch of the ANS is accompanied by an inhibition of the other. However, the sympathetic and parasympathetic branches also activate independently; thus, co-activation and co-inhibition may occur, which is demonstrated by a two-dimensional model of ANS activity. Here, we apply such models to assess how markers of the autonomic space relate to several critical psychological constructs: emotional contagion (EC), general anxiety, and positive and negative affect (PA and NA). We also examined gender differences in those psychophysiological relations.

**Methods:**

In the present study, we analyzed data from 408 healthy students, who underwent a 5-min group baseline period as part of their participation in several experiments and completed self-reported questionnaires. Electrocardiogram (ECG), electrodermal activity (EDA), and respiration were recorded. Respiratory sinus arrhythmia (RSA), pre-ejection period (PEP), as well as cardiac autonomic balance (CAB) and regulation (CAR) and cross-system autonomic balance (CSAB) and regulation (CSAR), were calculated.

**Results:**

Notably, two-dimensional models were more suitable for predicting and describing most psychological constructs. Gender differences were found in psychological and physiological aspects as well as in psychophysiological relations. Women's EC scores were negatively correlated with sympathetic activity and positively linked to parasympathetic dominance. Men's PA and NA scores were positively associated with sympathetic activity. PA in men also had a positive link to an overall activation of the ANS, and a negative link to parasympathetic dominance.

**Discussion:**

The current results expand our understanding of the psychological aspects of the autonomic space model and psychophysiological associations. Gender differences and strengths and weaknesses of alternative physiological models are discussed.

## 1 Introduction

### 1.1 Autonomic nervous system

The ANS has an established role in psychophysiological research (Cacioppo et al., 2007). Most studies that have examined how ANS activity is related to psychological functions investigated discrete measures of the ANS quantifying either activity of the sympathetic (SNS) or the parasympathetic (PNS) branch (or their combined activity). PNS control is theorized to reflect our capacity to flexibly respond to changes in environmental cues and stressors (Thayer and Lane, 2000; Porges, 2007), including the ability to regulate emotions (Thayer and Lane, 2000; Beauchaine, 2015; Balzarotti et al., 2017; Pace-Schott et al., 2019). SNS governs a wide array of visceral functions, and, in situations of threat and stress, triggers the activation of both the cardiovascular and adrenal catecholamine systems, facilitating a fight-or-flight response (Jansen et al., 1995; Sapolsky et al., 2000; Obradović and Boyce, 2012). SNS is thought to reflect heightened arousal during behavioral inhibition and avoidant coping (Dawson et al., 2007; Obradović and Boyce, 2012), which are greatly linked to anxiety disorders (Hirschfeld, 2001).

There are several indices of ANS function, which have been extensively studied in psychological research in the past. Respiratory sinus arrhythmia (RSA) is one aspect of the coupling between the cardiac and the respiratory systems (Bartsch et al., 2014). It indicates periodic variations of the heart rate within a breathing cycle and is considered an indicator of cardiac PNS activity (Cacioppo et al., 1994; Malik et al., 1996; Berntson et al., 1997; Bartsch et al., 2012). Low resting RSA has been linked in several studies to anxiety disorders (Friedman and Thayer, 1998; Friedman, 2007; Chalmers et al., 2014), while high levels of resting RSA are considered to indicate more integration between peripheral and central nervous activity which allows for more flexible responses and greater emotional regulation (Thayer and Lane, 2000; Porges, 2007; Beauchaine, 2015; Balzarotti et al., 2017; Pace-Schott et al., 2019). Electrodermal activity (EDA), an indicator of the SNS that reflects activity of the eccrine sweat glands driven by cholinergic neurotransmission, has been widely studied in the context of anxiety disorders (Fowles, 1986; Shields et al., 1987; Dawson et al., 2007). Resting EDA levels, as well as EDA measured in a variety of settings, have been consistently related to anxiety (for reviews see: Hoehn-Saric and McLeod, 1988. Also: Balyan et al., 2016; Barlow, 2002; Boucsein, 2012). Another marker of SNS activity is pre-ejection period (PEP), defined as the length of time between an electrical signal reaching the left ventricle of the heart and indicating it to contract and the time when the aortic valve opens (Sherwood et al., 1990; Berntson et al., 1994a; Cacioppo et al., 1994; Benevides and Lane, 2013; Stone et al., 2020). PEP is highly predictive of cardiac sympathetic control (Cacioppo et al., 1994) and is thought to be linked to active coping (Brenner et al., 2005; Kelsey, 2012; Obradović and Boyce, 2012) and to stress sensitivity (Beauchaine, 2001). The association between PEP and anxiety is not consistent, with different studies reporting positive correlations (Pollatos et al., 2007), negative correlations (Kossowsky et al., 2012; Fu et al., 2018) or no associations at all (Burns et al., 1992; Sperry et al., 2018). RSA, EDA and PEP are all distinct indices of ANS, each indicating discrete aspects of the functioning of the ANS.

Due to evidence suggesting that the two branches of the ANS can activate independently (Berntson et al., 1991; Berntson and Cacioppo, 2007; Stone et al., 2020), the autonomic balance and regularity capacity model was offered to describe the full range of autonomic patterns that may occur (i.e., co-activation or co-habitation of the PNS and SNS, Berntson et al., 1991, 1993, 1994b; Berntson and Cacioppo, 2007). Cardiac autonomic balance (CAB) and cross-system autonomic balance (CSAB) quantify the differences between PNS (measured by RSA) and SNS (measured by PEP or EDA, respectively) activities and represent the relative influences of the ANS branches, with lower scores indicating SNS dominance and higher scores indicating PNS dominance. Cardiac autonomic regulation (CAR) and cross-system autonomic regulation (CSAR) represent the sum of PNS and SNS activities, and constitute markers of overall autonomic activation (i.e., co-activation or co-inhibition) of both PNS and SNS branches (Berntson et al., 2008; Stone et al., 2020). Since PEP and EDA capture distinct aspects of the SNS and their activities are not necessarily aligned (see, for example, Brenner et al., 2005; Kreibig et al., 2007; Stone et al., 2020), CAB, CAR, CSAB and CSAR may produce different representations of the ANS activity. While some studies have linked CAB, CAR, CSAB and CSAR to health problems (Berntson, 2019; Alen et al., 2020), anxiety symptoms (Stone et al., 2020) and psychopathologies (Bylsma et al., 2015; Brush et al., 2019; Cohen et al., 2020; Stone et al., 2020), only a few studies to date have examined the relationship between those indices at rest or baseline and several meaningful psychological functions in a healthy adult population.

### 1.2 Psychological indices

In the current study we focused on four psychological indices which have a high influence on social interaction.

#### 1.2.1 Emotional contagion

EC is the automatic tendency to catch another's emotions (Hatfield et al., 1992, 1993; Mayo et al., 2023) and can be manifested as a demonstration of similar postural, vocal, or facial expression or as similar neurophysiological or neurological patterns of activity (Schoenewolf, 1990; Hatfield et al., 1994, 2009, 2014; Barsade, 2002; Barsade et al., 2018). EC is related to empathy, attunement, and bonding (Hatfield et al., 1994, 2011; Spoor and Kelly, 2004; Decety and Ickles, 2009; Neves et al., 2018), as well as to stress (Feldman and Kaal, 2007), and has been suggested to facilitate social interactions (Hatfield et al., 1994; Butler, 2011). Individuals differ in their susceptibility to EC (Doherty, 1997; Hatfield et al., 2014; Barsade et al., 2018; Horesh et al., 2021), and women usually score higher than men in EC performance and questionnaires (Hall, 1978; Buck, 1984; Doherty et al., 1995; Surakka and Hietanen, 1998; Wild et al., 2001; Sonnby-Borgström et al., 2008; Magen and Konasewich, 2011; Manera et al., 2013; Fairbairn et al., 2015).

Associations between EC and physiological synchrony had been suggested and demonstrated many times (see Table 1 for details), yet the connection between EC as a trait and baseline physiological markers of the ANS remain largely unknown.

**Table 1:** This table details previously found associations between EC and physiological synchrony.

**Psychophysiological relations with EC**				
**Physiological markers**	**Research population**	**Task or setting**	**Main findings**	**References**
Heart rhythm pattern	64 undergraduate students	A facial expression imitation task	EC significantly increased physiological correlation between the participants.	Park et al., 2019
RSA, inter-beat interval, PEP	98 mother-infant dyads	Playing time with or without touch	Infants' physiological responses were influenced by the mothers' affective states and by the touch condition.	Waters et al., 2017
PEP, heart rate	69 mother-infant dyads	A relaxation interaction after manipulation of mothers' stress levels	Infants' physiological synchrony with their mothers, as well as their social behavior, were influenced by the stress manipulation experienced by the mothers.	Waters et al., 2014
Cortisol level	30 married couples	measurements were taken 4 times a day for 3 days	Spouses' physical proximity strengthen both physiological synchrony and EC of negative moods.	Saxbe and Repetti, 2010
Inter-beat interval	21 performers and 63 watchers	Participants watched a video interaction of varying levels of stress	Observers' physiological changes were related to the speaker's stress level.	Dimitroff et al., 2017

Each line specifies one research and includes information regarding the physiological markers used, the research population, the tasks performed and the main relevant findings.

#### 1.2.2 Positive affect and negative affect.

PA and NA are highly distinctive and almost orthogonal dimensions of affective structure (Russell, 1980, 1983; Stone, 1981; Zevon and Tellegen, 1982; Watson et al., 1984; Diener et al., 1985; Clark and Watson, 1988) and are commonly collected via self-reported mood questionnaires in psychological research (Clark and Watson, 1988; Clark et al., 1989; Futterman et al., 1992; Christie and Friedman, 2004; Fredrickson and Losada, 2005; Lai et al., 2005; Pressman and Cohen, 2005; Kubzansky and Thurston, 2007; Nabi et al., 2008; Oveis et al., 2009; Steptoe et al., 2009; Dockray and Steptoe, 2010; Drachen et al., 2010; Cribbet et al., 2011; Diamond et al., 2011; Shiota et al., 2011; Wang et al., 2013). PA represents feelings of enthusiasm, activeness, and alertness, with high PA being a state of high energy, concentration, and pleasurable engagement whereas low PA is a state of sadness and lethargy (Clark and Watson, 1988). NA reflects distress and unpleasurable engagement and includes a variety of aversive mood states, such as anger, contempt, disgust, guilt, fear, and nervousness, with low NA being a state of calmness and serenity (Clark and Watson, 1988). Both PA and NA are usually higher in women compared to men (for a review see Batz and Tay, 2018, and also: Fujita et al., 1991; Grossman and Wood, 1993; Thomsen et al., 2005; Zuckerman et al., 2017).

Many studies have examined the sympathetic and parasympathetic responses to PA (see Table 2) and to NA (see Table 3), with conflicting results. Thus, an examination of autonomic space model markers might be more suitable to describe the physiological manifestation of those important psychological constructs.

**Table 2:** This table details previously found associations between PA and physiological activity.

**Psychophysiological relations with PA**				
**Physiological markers**	**Research population**	**Task or setting**	**Main findings**	**References**
Inter-beat interval, EDA, facial electromyography	37 men	The Rorschach test	Positive correlation between PA and parasympathetic activity	Kettunen et al., 2000
Heart rate variability, electroencephalo-graphy	20 adults	Event recall tasks and Stroop Color Word Test	Positive correlation between PA and parasympathetic activity	Kop et al., 2011
Heart rate variability	18 adults	Video game playing in Pairs.	Positive correlation between PA and parasympathetic activity	Noah et al., 2015
RSA	80 students	Emotion-inducing videos watching	Positive correlation between PA and parasympathetic activity	Oveis et al., 2009
RSA	98 students	Affect reporting 3 times in 1 year	Positive correlation between PA and parasympathetic activity	Wang et al., 2013
Systolic and diastolic blood pressure, heart rate, PEP, stroke index, cardiac index, total peripheral resistance	42 students	Recall tasks	heightened sympathetic activity correlated with higher PA scores	Neumann and Waldstein, 2001
Heart rate, RSA, PEP, facial electromyography	77 adults	Startle, mental arithmetic, reaction time, and speech tasks.	PA was associated with a sympathetic reactivity and with a parasympathetic withdrawal in all tasks among women, and during the speech task among men.	Heponiemi et al., 2006

Each line specifies one research and includes information regarding the physiological markers used, the research population, the tasks performed and the main relevant findings.

**Table 3:** This table details previously found associations between NA and physiological activity.

**Psychophysiological relations with NA**				
**Physiological markers**	**Research population**	**Task or setting**	**Main findings**	**References**
Inter-beat interval, skin conductance, general somatic activity, pulse transmission time to the finger, finger pulse amplitude, finger temperature, pulse transmission time to the ear	151 married couples	Three conversations and silent periods	For husbands, NA was correlated with physiological activity during a conflict and a pleasant topic conversation. No relation was found for wives or for a neutral conversation.	Levenson et al., 1994
EDA, heart rate variability	18 adults	Virtual reality scenes watching after mental stress	Natural scenes reduced both sympathetic activity and NA more than the control scene.	Anderson et al., 2017
EDA, heart rate	16 participants	Video game playing	A positive correlation between NA and sympathetic activity	Drachen et al., 2010
RSA, skin conductance, number of electrodermal responses	94 students	Watching of positive or negative short movie	No connection was found between physiology and self-reported affect. Higher baseline RSA was associated with less negative facial affect in response to the negative film.	Demaree et al., 2006
RSA, PEP	87 children	Quietly sitting, coloring, and reading a book	No connection was found between ANS activation at rest and NA.	Zhou et al., 2022
RSA	80 students	Emotion-inducing videos watching	No connection was found between RSA at rest and NA.	Oveis et al., 2009
RSA	98 students	Affect reporting 3 times in 1 year	No connection was found between resting RSA and NA.	Wang et al., 2013
Heart rate, RSA, PEP, facial electromyogr-aphy	77 adults	Startle, mental arithmetic, reaction time, and speech tasks	No connection was found between NA and sympathetic arousal.	Heponiemi et al., 2006

Each line specifies one research and includes information regarding the physiological markers used, the research population, the tasks performed and the main relevant findings.

#### 1.2.3 Anxiety

Anxiety is a trait depicting individuals' tendency to regard many situations as dangerous or alarming and to respond to them accordingly (Spielberger, 1966; Elwood et al., 2012; Sege et al., 2018). Anxiety is also a state of ANS arousal in which a person consciously experiences feelings of tension and worry (Spielberger, 1966). Women are usually reported to have higher levels of anxiety than men (Angst and Dobler-Mikola, 1985; Weissman and Merikangas, 1986; Anderson et al., 1987; Bourdon et al., 1988; Líndal and Stefánsson, 1993; Campbell and Rapee, 1994; Kessler et al., 1994, 1995; Lewinsohn et al., 1998; Poulton et al., 2001; Muris and Ollendick, 2002; Costello et al., 2003; Bruce et al., 2005; McLean et al., 2011).

State anxiety had been extensively studied in the context of ANS activity (see Table 4 for details), yet the relation between state anxiety and sympathetic and parasympathetic activities is complex.

**Table 4:** This table details previously found associations between state anxiety and physiological activity.

**Psychophysiological relations with state anxiety**				
**Physiological markers**	**Research population**	**Task or setting**	**Main findings**	**References**
Heart rate, heart rate variability	52 adults	Filling in 1 day of stress diary	State anxiety was positively correlated with sympathetic activity and negatively correlated with parasympathetic activity.	Brosschot et al., 2007
Heart rate, finger pulse volume, skin conductance	45 women	A speech or a written narration task	A positive correlation was found between state anxiety and sympathetic activity.	Gonzalez-Bono et al., 2002
Heart rate	17 students	Giving a presentation and watching another student's presentation	A positive correlation was found between state anxiety and sympathetic activity.	Kantor et al., 2001
Cortisol level, prolactin level, noradrenaline level, adrenaline level	38 men	Measurements at baseline and before exams	Sympathetic activity was higher before the test, but no correlations were noted between hormonal changes and state anxiety.	Herbert et al., 1986
Finger pulse volume	62 students	Measurements before and during an interview.	A positive correlation between state anxiety and sympathetic activity.	Smith et al., 1984
Heart rate, EDA, skin temperature	29 children with or without autism spectrum disorder diagnosis	Movie watching or taking the Stroop task	A positive correlation between state anxiety and sympathetic activity was found in both groups.	Kushki et al., 2013
Heart rate variability, inter-beat intervals	63 high or low trait anxiety individuals	Stress inducing task or a relaxation session	Stress condition affected sympathetic activity.	Miu et al., 2009
EDA	50 eighth grade students	A mathematics test	The researchers divided anxiety into different components. Motivational component was positively correlated and the cognitive and affective components were negatively correlated with EDA.	Roos et al., 2022
Heart rate, heart rate variability	52 adults	Filling in a stress diary for 1 day	Stressors and worry were linked to a higher sympathetic activity and to a parasympathetic withdrawal.	Brosschot et al., 2007
RSA, inter-beat intervals	45 women	Measurements taken at 3 time points in the span of 3 weeks	A positive correlation between state anxiety and sympathetic activity.	Fuller, 1992
RSA	82 adults	Measurements were taken at baseline	A positive correlation between state anxiety and parasympathetic activity.	Jönsson, 2007
Heart rate variability, baroreflex sensitivity	86 hypersensitive and 98 control individuals	A tilt table examination	No correlation was found between state anxiety and physiological function.	Bajkó et al., 2012
RSA	20 adults	A non-word reading task and preparation and delivery of a speech	No correlation was found between state anxiety and physiological function.	Bauerly and Jones, 2021
PEP, RSA	97 students	Emotion regulation condition and a speech task	No correlation was found between state anxiety and physiological function.	Cho et al., 2019
Heart rate, cortisol level, secretory immunoglobulin A	58 students	An examination	No correlation was found between state anxiety and physiological function.	Huwe et al., 1998
Cortisol level	92 students	An examination	No correlation was found between state anxiety and physiological function.	Ringeisen et al., 2019
Cortisol level	65 students	An examination	No correlation was found between state anxiety and physiological function.	Spangler et al., 2002
Heart rate, root mean square of successive differences of inter-beat intervals	60 adults	A speaking stressor or a control condition	No correlation was found between state anxiety and physiological function.	Verkuil et al., 2014
RSA, EDA, PEP, CAB, CAR, CSAB, CSAR	85 women	Measurements were collected at baseline	Higher anxiety symptoms were associated only with lower CSAB scores.	Stone et al., 2020

Each line specifies one research and includes information regarding the physiological markers used, the research population, the tasks performed and the main relevant findings.

### 1.3 Gender differences

#### 1.3.1 Psychological gender differences

Gender differences had been the focus of intense investigation and interest for more than a century, with research consecrating on various aspects of psychology, such as cognitive abilities, personality and social behaviors (for reviews see Hyde et al., 1990 and Hyde, 2014).

The psychological indices which interested us in the current study were also examined for gender differences in the literature. Specifically, gender differences had been widely studied in anxiety. Although women report more anxiety symptoms and have a greater risk than men to develop most kinds of anxiety disorders (Angst and Dobler-Mikola, 1985; Weissman and Merikangas, 1986; Anderson et al., 1987; Bourdon et al., 1988; Líndal and Stefánsson, 1993; Campbell and Rapee, 1994; Kessler et al., 1994, 1995; Lewinsohn et al., 1998; Poulton et al., 2001; Muris and Ollendick, 2002; Costello et al., 2003; Bruce et al., 2005; McLean et al., 2011), there appears to be no difference in baseline state anxiety between the genders (Carrillo et al., 2001; Brand and Schoonheim-Klein, 2009; Limbu et al., 2010; Strohmaier et al., 2020). In regard to EC, it had previously been suggested that women are better than men at reading the emotional displays of other individuals (Haviland and Malatesta, 1981; Hall, 1984), which may facilitate their ability to emphasize and mimic the emotional states of others (Hatfield et al., 1992). Indeed, EC in women is time and again being reported as higher than men's EC, under various conditions (Hall, 1978; Buck, 1984; Doherty et al., 1995; Surakka and Hietanen, 1998; Wild et al., 2001; Sonnby-Borgström et al., 2008; Magen and Konasewich, 2011; Manera et al., 2013; Fairbairn et al., 2015). Affective states may also be influenced by gender. Women are usually reported to experience higher levels of PA (for a review see Batz and Tay, 2018) and of NA (Fujita et al., 1991; Grossman and Wood, 1993; Thomsen et al., 2005; Zuckerman et al., 2017) than men, although some studies found no gender differences in levels of affect (Neumann and Waldstein, 2001; Cribbet et al., 2011; Volante et al., 2016).

#### 1.3.2 Gender differences in ANS activity

Over the past few decades some evidence emerged suggesting that gender differences may exist in ANS activity (for reviews see Hinojosa-Laborde et al., 1999; Dart et al., 2002; Pothineni et al., 2016). Most studies found no differences in parasympathetic dominance in the form of high-frequency heart rate variability (HRV) or RSA between men and women (Frazier et al., 2004; Codispoti et al., 2008; Cribbet et al., 2011; Moodithaya and Avadhany, 2012; Botek et al., 2018).

Regarding SNS activity, however, the picture is more complicated (see Table 5 for details).

**Table 5:** This table presents previous findings regarding gender differences in sympathetic physiological activity, specifically in measures of EDA, skin conductance, heart rate and PEP.

**Sympathetic Differences Between Men and women**				
**Physiological markers**	**Research population**	**Task or setting**	**Main findings**	**References**
EDA	53 women and 33 men	Mathematical test	Higher levels of EDA activity were noted for men.	Strohmaier et al., 2020
EDA	30 women and 30 men	Relaxation and mathematical test	No gender differences were found.	Bari, 2020
EDA	28 women and 28 men	Baseline and emotion eliciting film viewing	No gender differences were found.	Frazier et al., 2004
EDA	18 women and 18 men	Exposure to visual, cognitive, and breathing stimuli, as well as to three different temperatures	EDA differed between the genders only under conditions of high temperatures.	Qasim et al., 2022
Skin conductance	33 women and 27 men	Viewing of highly arousing pleasant and unpleasant films	No gender differences were found.	Codispoti et al., 2008
Skin conductance	50 women and 45 men	Emotion eliciting picture viewing	Men had higher EDA reactivity only to pleasant stimuli.	Bradley et al., 2001
Skin conductance	54 women and 41 men	Exposure to disgust-content pictures	Women had a higher EDA response to disgust.	Rohrmann et al., 2008
Skin conductance	640 participants aged 5–25 of both genders	Physiological measures during different seasons and times of day	Season and time of day influenced only women's physiology, which was generally higher than the men's.	Venables and Mitchell, 1996
Heart rate	62 women and 68 men	Rest	Women presented higher heart rate than men.	Flanagan et al., 2007
Heart rate	615 women and 603 men	Mega analysis of data from 20 groups	Women presented higher heart rate than men.	Koenig et al., 2021
Heart rate	36 women and 41 men	Startle, mental arithmetic, reaction time, and speech tasks	Women presented higher heart rate reactivity than men in response to stress.	Heponiemi et al., 2006
Heart rate	141 females and 126 males, ages 6–55	Rest	Women presented unsignificant higher heart rate than men.	Moodithaya and Avadhany, 2012
Heart rate	135 women and 141 men	A 24-h Holter recording	Heart rate was higher for men until the age of 40.	Ramaekers et al., 1998
Heart rate, PEP	22 women and 20 men	A nonverbal math task, a mirror tracing task, the Stroop Color-Word interference task, and an isometric handgrip task	Women presented higher heart rate reactivity than men in response to the mirror tracing and the Stroop tasks. No gender differences were found for PEP.	Allen et al., 1993
Heart rate, PEP	62 women and 32 men	Mental stress laboratory task	No gender differences were found.	Brydon et al., 2012
Heart rate, PEP	5,026 subjects, ages 7–17	A meta-analysis of stress responses to the Trier Social Stress Test	No gender differences were found.	Seddon et al., 2020
Heart rate, PEP	22 women, 20 men	Recall tasks	No gender differences were found.	Neumann and Waldstein, 2001

Each line specifies one research and includes information regarding the relevant physiological markers used, the research population, the tasks performed and the main relevant findings.

### 1.4 Current study

#### 1.4.1 Research goals and contributions

Considering the above, the main goal of the current study was to determine whether a two-dimensional autonomic space approach is preferable to the usage of a single physiological measure in assessing the relation between markers of ANS activity and several important aspects of psychological function in healthy adults during a social baseline period. A secondary aim was to estimate gender differences in the manifestation of those psychophysiological correlations.

We focused on four psychological constructs, EC, PA, NA and state anxiety, all of which are highly influential on social interaction, and are widely studied in psychological literature in general and in the area of gender differences in particular. However, to our knowledge, only scant research (Stone et al., 2020) has examined CAB, CAR, CSAB or CSAR associations with anxiety, while PA, NA and EC have not yet been extensively tested in the context of the autonomic space models.

While gender differences in individual psychological functions had been extensively studied and physiological indices were also assessed for gender diversities in past research, variations between men and women in psychophysiological correlations are largely unknown. In the present study we aspired to bridge that knowledge gap as well as to further examine gender differences at the level of individual psychological and physiological indices.

#### 1.4.2 Gender differences and psychophysiological hypotheses for EC

EC is robustly reported as being higher in women compared to men, and we expected to find the same in our research. The link between EC as a trait and physiological activity, however, is largely unknown and was explored here with the autonomic space model for the first time.

#### 1.4.3 Gender differences and psychophysiological hypotheses for PA and NA

Since both PA and NA are usually reported to be higher in women than in men, we hypothesized women to score higher than men in both dimensions of affect. Regarding physiological activities, most studies link PA to increased PNS activity in general and specifically to rise in RSA and some associate PA with sympathetic activity. We, therefore, hypothesized that PA would be positively correlated with RSA, EDA, PEP, CAR and CSAR, meaning that individuals who display higher PA scores will be characterized by higher levels of sympathetic, parasympathetic, and total ANS activation. Since more studies indicate higher PNS activity rather than SNS activity in association with higher PA, we speculated PA to positively correlate with CAB and CSAB, indicating parasympathetic dominance.

NA was usually associated in past research with sympathetic activity and parasympathetic withdrawal. Hence, we hypothesized NA to be positively correlated with EDA and PEP and negatively associated with RSA, meaning that participants with higher NA scores will demonstrate higher sympathetic and lower parasympathetic activities. We also assumed individuals with higher NA scores will have lower CAB and CSAB scores, indicating sympathetic dominance. The correlations between CAR and CSAR and NA had never been tested before, and we therefore had no prior hypothesis regarding them and examined those connections here for the first time.

#### 1.4.4 Gender differences and psychophysiological hypotheses for state anxiety

The literature shows that women are more anxious and are more likely to develop an anxiety disorder than men. However, when measuring baseline levels of state anxiety, no gender differences were found in previous research. We therefore expected no gender differences in our measurements of baseline state anxiety.

The relations between state anxiety and physiological activities are complex. While the majority of studies indicate a positive correlation between state anxiety and sympathetic activity, the relations state anxiety has with PEP at rest are either negative or non-existent. RSA is usually reported to withdraw with a rise in state anxiety. We therefore hypothesized a positive correlation between state anxiety and EDA and a negative correlation with RSA and PEP.

CAB, CAR, CSAR, and CSAB had only been examined once in relation to anxiety (Stone et al., 2020). CSAB had a negative correlation with state anxiety, and this was the correlation we expected to find in our study, while for CAB, CAR, and CSAR there were no correlations detected. We could therefore produce no prior hypotheses concerning those three physiological indices.

#### 1.4.5 Gender differences in physiological indices

Most studies to date report no gender differences in parasympathetic activity as measured by RSA or in PEP representation of sympathetic dominance. Although EDA reactivity is stronger in men, no gender differences were observed at rest. We therefore expected men and women to express roughly the same levels of all types of physiological indices. Table 6 summarizes the entirety of our hypotheses regarding psychophysiological relations (Table 6A) as well as gender differences in the distributions of psychological scores and physiological measures (Table 6B).

**Table 6A:** A summary of all hypotheses regarding psychophysiological correlations, based on past research.

**Indices**	**EC**	**PA**	**NA**	**State anxiety**
RSA	?	+	–	–
EDA	?	+	+	+
PEP	?	+	+	–
CAB	?	+	–	?
CAR	?	+	?	?
CSAB	?	+	–	–
CSAR	?	+	?	?

Psychological constructs are presented at the top line of each column while physiological indices are written at the left column of the table. + represents an anticipated positive psychophysiological correlation while hypotized negative relation is indicated by –. Associations we could not predict based on the literature are marked by ?.

**Table 6B:** A summary of anticipated gender differences in psychological and physiological markers.

**Women dominance**	**Men dominance**	**No gender difference**
EC, PA, NA		State Anxiety, RSA, EDA, PEP, CAB, CAR, CSAB, CSAR

Indices expected to be higher in women are at the bottom left cell, the bottom middle cell portrays the indices for which men were hypothesized to score higher, and psychological and physiological indicators for which no gender difference was anticipated are at the bottom right cell.

## 2 Method

### 2.1 Participants and procedure

Data was collected from four studies recently performed at the Social Neuroscience lab at Bar-Ilan University, Israel: (A) a study aimed to examine associations between physiological and behavioral synchrony to group cohesion and performance (Gordon et al., 2020a,b); (B) a study aimed to inspect the influence of political rivalry on group processes via the role of intermediate physiological mechanisms; (C) a study aimed to investigate physiological reactions to justice, and (D) a pilot study for study C (Gordon et al., 2021). After providing informed consent, all studies began with a 5-min social baseline procedure in which groups of three participants were asked to sit down quietly together with their eyes open, to not look at each other but to choose a location or object in the room and look toward it and not do anything else for a 5-min resting physiological recording period. We used the resting physiological data from the initial baseline period in the current study.

Undergraduate students (*n* = 498) studying at the Department of Psychology, Bar-Ilan University, participated in these studies. Study A included 145 participants (30 men, mean age = 22.51 years, SD = 2.1); 144 subjects participated in Study B (51 men, mean age = 23.43 years, SD = 3.39), study C involved 149 participants (50 men, mean age = 24.09 years, SD = 3.98), and 60 individuals took part in study D (16 men, mean age = 22.96 years, SD = 2.43). Ninety participants were removed from the physiological analysis (*n* = 41 from study A, *n* = 28 from study B, *n* = 17 from study C, and *n* = 4 from study D): *n* = 56 participants had incomplete physiological recordings, and *n* = 34 participants had unreliable recordings [e.g., heart rate was below 40 beats per minute (bpm) or above 110 bpm, or the breathing frequency was below 0.01 Hz or above 0.5 Hz, corresponding to breathing rates of 0.6 and 30 breaths per minute, respectively]. Overall, data from 408 participants were analyzed.

As can be seen, there is a large gap between the number of men and women participating in the study. We were limited to influence the composition of the study population due to the nature of this research, which is based on data previously gathered in past studies. However, any statistical analysis should be carefully considered with this imbalance in mind. Please see the discussion on this subject.

All participants provided written informed consent in accordance with the request of the University's Institutional Review Board (IRB). All procedures of the studies were done based on the ethical guidelines of the IRB approvals.

### 2.2 Physiological data

In all studies, participants were individually wired to a MindWare Mobile recording unit (MindWare Technologies LTD, Westerville, OH, USA), a well-validated and specialized hardware and software system for monitoring cardiac performance, autonomic balance, and respiratory activity. The recorder enabled, via seven electrodes, the measurement of electrocardiogram (ECG) (through which HRV can be derived), impedance cardiographic waveforms (enabling PEP calculation) and electrodermal activity (EDA).

ECG was recorded at a sampling rate of 500 Hz using a standard lead II configuration. It was analyzed with the MindWare Technology's HRV application software (version 3.1.4) as a single segment. The ECG signal was amplified by a gain of 1,000 and filtered with a hamming windowing function. The data was then visually inspected and manually edited by trained graduate and undergraduate students to ensure proper removal of artifacts and ectopic beats (Berntson et al., 1997).

RSA scores were calculated for each baseline period as a one 5-min segment using MindWare's HRV application. The procedure of determining RSA entails the detection of R-peaks (i.e., heartbeats) in the clean ECG recording and the calculation of an interbeat-interval (IBI) time series. This time series is resampled to 2 Hz (by 500 ms interpolation) in order to obtain equidistant IBI and detrended by removing the global linear trend. The resampled and detrended IBI time series is then analyzed by Fast Fourier Transform (FFT) (using a Hamming window function) to calculate the Heart Period Power Spectrum. MindWare automatically chooses a high frequency (HF) band—a range of 0.12–0.4 Hz (for 15.8% of the participants in our database) or 0.12–0.42 Hz (for 84.2% of participants). We used the respiration time series to verify that the average respiration frequency falls into the selected HF band. Finally, the spectral power of the HF band is calculated, and its natural logarithm equals the RSA score for that given baseline period. PEP scores were calculated as an average for the entire baseline period using the clean and filtered ECG as well as impedance waveforms, with Mindware Impedance Cardiographic software module. PEP is quantified as the time interval between the electrical invasion of the ventricular myocardium (Q-wave of the ECG) and the opening of the aortic valve (B point of the impedance waveform (dZ/dt), Sherwood et al., 1990). The Q-wave peak was automatically marked as the point in which the maximal change in slope occurred during the 35-milliseconds preceding the R-wave peak in the ECG record.

Skin conductance was obtained at a sampling rate of 500 Hz through two Ag/AgCl MindWare electrodes, placed on the non-dominant palm of each individual. It was analyzed using Mindware Technology's EDA application software (version 3.1.5) and visually inspected and manually edited by trained graduate students to ensure proper removal of artifacts. The skin conductance signal was smoothed with a rolling filter of 500 data points per block. For every 500 milliseconds, the level of skin conductance was outputted in MicroSiemens (threshold of 0.05). EDA was calculated as the mean of skin conductance scores in the entire recording period (Picard et al., 2016).

### 2.3 Autonomic space model calculations

In order to calculate CAB, CAR, CSAB and CSAR we first calculated *z*-scores for all RSA, PEP and EDA values of all 408 participants, using MATLAB. CAB scores were calculated as the differences between subjects' RSA and PEP scores, while CAR equaled RSA and PEP's sums. CSAB was calculated as the individual's RSA *z-*score minus his or hers EDA *z*-score. CSAR was calculated as the sum of those *z*-scores.

### 2.4 Self-reported measures

Prior to baseline measurements, all participants were asked to complete several questionnaires. We focus here on three such measures detailed below. The Emotional Contagion Scale (Doherty, 1997) and a mood questionnaire (Watson et al., 1988) were included in all four studies. A short version of the General Anxiety questionnaire (Marteau and Bekker, 1992) was included only in studies C and D. In the entire dataset we analyzed for this report, 400 individuals reported on the Emotional Contagion scale, 404 individuals reported on their mood via the PANAS questionnaire, and 197 individuals rated General Anxiety. Missing data is due to the fact that not all individuals who participated in the study fully completed their self-report questionnaires. Furthermore, 404 of all participants reported on their gender.

#### 2.4.1 Emotional contagion scale

The EC scale is a well-established 15-item measure of individual susceptibility to the emotions of others (Doherty, 1997). This trait-like measure estimates mimetic tendency to five basic emotions (love, happiness, sadness, fear, and anger), derived from afferent feedback caused by mimicry. There is a positive association between susceptibility and emotionality, self-esteem, empathy, affective orientation and sensitivity to others, and a negative correlation with emotional stability, alienation, and self-assertiveness. A higher score indicated a higher self-reported tendency to “catch” emotions. In the current sample, EC's Cronbach's alpha was 0.893.

#### 2.4.2 Positive and negative affect schedule

PANAS is a reliable, brief, and valid mood scale that measures the state of two primary dimensions of mood—positive and negative affect (Watson et al., 1988). PA indicates how enthusiastic, alert, and active a person feels, with low positive affect being a state of sadness and apathy. PA is reflective of concentration, pleasurable engagement, and high energy, and is related to social activity and satisfaction. NA refers to subjective distress and unpleasurable engagement accompanied by unpleasant mood states, such as fear, disgust, anger, contempt, and nervousness. NA is associated with self-reported stress and health complaints, while low NA reflects a state of serenity and calmness. Both positive and negative dimensions are largely uncorrelated, exceedingly internally consistent, and quite stable over a 2-month period. A higher score in each dimension indicated higher reports of positive or negative mood. We used a 20-item version of PANAS in studies A and B (Cronbach's alpha of 0.853) and a 25-item version of the PANAS questionnaire in studies C and D (Cronbach's alpha of 0.721).

#### 2.4.3 State anxiety

The Spielberger State-Trait Anxiety Inventory is a reliable, valid and one of the most widely used measure of state anxiety (Spielberger et al., 1970; Marteau and Bekker, 1992). It assesses the level of anxiety a person currently feels and is rather sensitive to fluctuations in transitory anxiety. A higher score indicated higher anxiety levels. Cronbach's alpha was 0.943 in our study.

### 2.5 Statistical calculations

**Figure 1:**
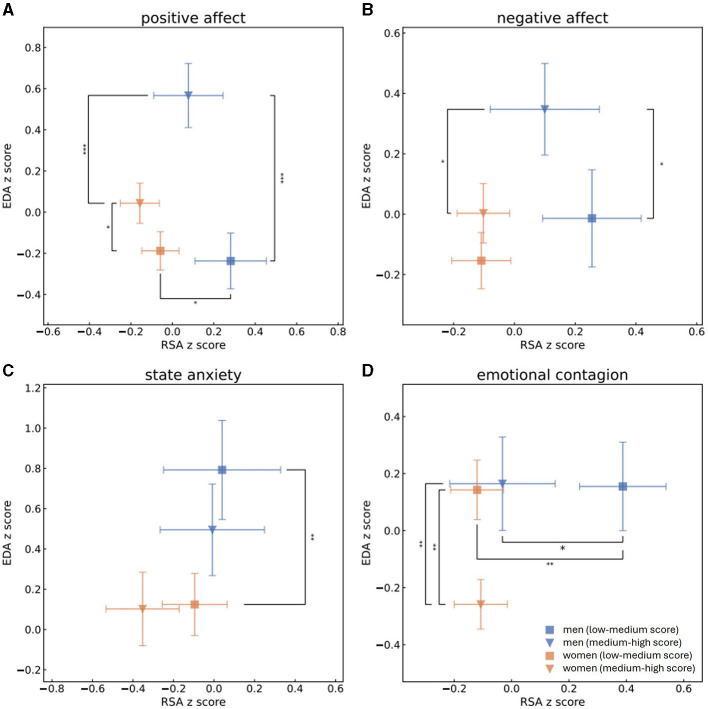
Mean and standard error values of the z-scores of RSA and EDA measurements of men with high (blue triangle) or low (blue square) scores and of women with high (orange triangle) or low (orange square) scores of PA **(A)**, NA **(B)**, state anxiety **(C)** and EC **(D)**. This grouping to high vs. low scores was determined by splitting the population to scores (s) of PA, NA, state anxiety or EC according to the median. One-sided *t*-tests were performed to determine whether men and women who received high and low scores of PA, NA, state anxiety and EC were separated from each other in terms of parasympathetic activity (RSA *z*-scores). Mann-Whitney *U-*tests were performed to determine group separation in terms of sympathetic activity (EDA *z*-scores). Statistical significance is marked by * for *p* < 0.05, ** for *p* < 0.01 and *** for *p* < 0.001. Men and women who reported on high PA levels experienced higher levels of EDA. Higher scores of PA in men were associated with higher levels of sympathetic activities than higher scoring women, while men who received low PA scores had higher levels of parasympathetic activities than low scoring women. Men who received higher scores of NA were characterized by higher levels of sympathetic activities than either men who had low NA scores of women who had high NA scores. Regarding individuals who had low scores of state anxiety, men were identified by higher sympathetic recordings than women. Women characterized by higher levels of EC experienced lower levels of sympathetic activity than either women identified by lower levels of EC or melas who had high EC scores. Men who received lower EC scores had higher parasympathetic activity then either men who scored higher EC scores or women who had lower scores of EC.

**Figure 2:**
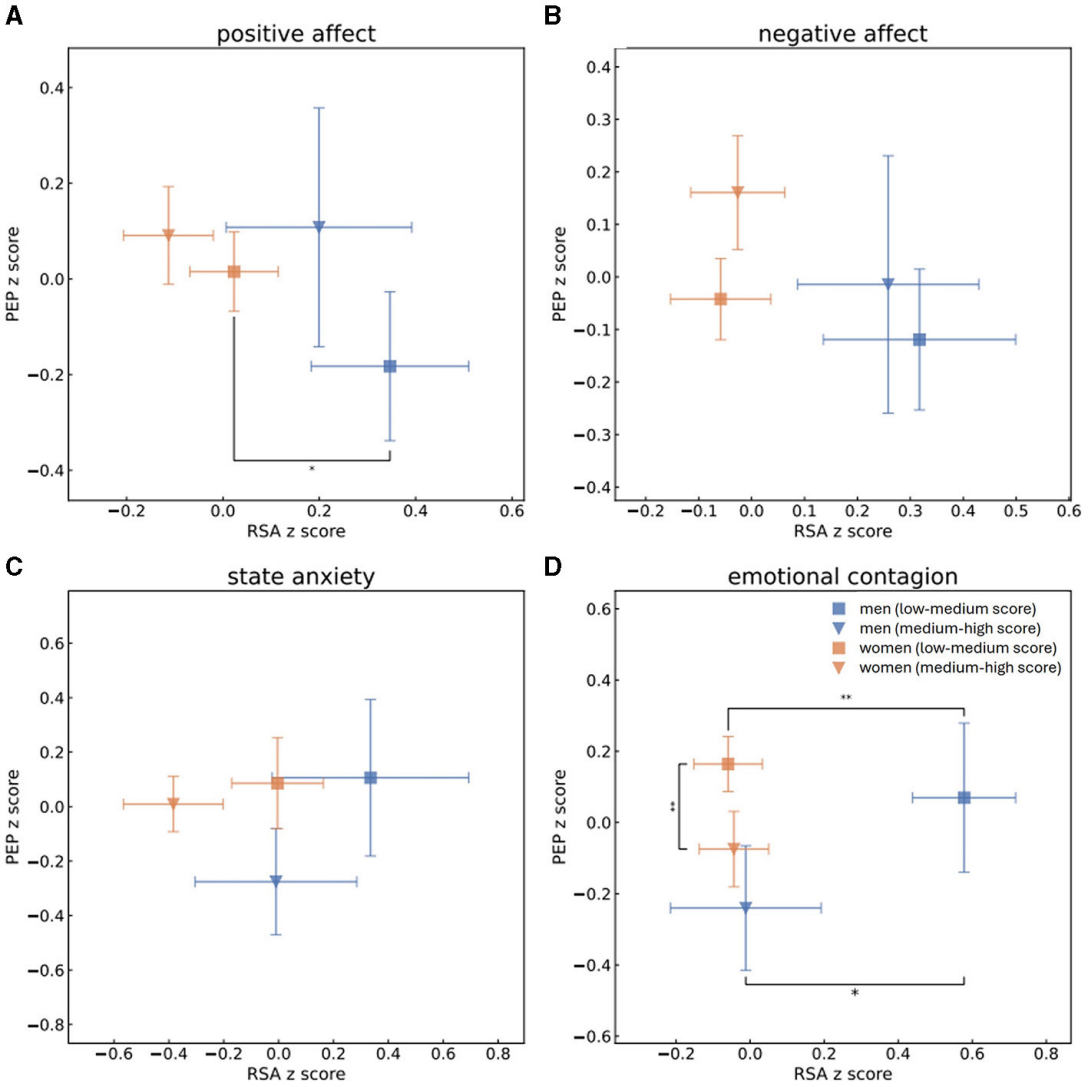
Mean and standard error values of the *z*-scores of RSA and PEP measurements of men with high (blue triangle) or low (blue square) scores and of women with high (orange triangle) or low (orange square) scores of PA **(A)**, NA **(B)**, state anxiety **(C)** and EC **(D)**. This grouping to high vs. low scores was determined by splitting the population to scores (s) of PA, NA, state anxiety or EC according to the median. One-sided *t*-tests were performed to determine whether men and women who received high and low scores of PA, NA, state anxiety and EC were separated from each other in terms of parasympathetic activity (RSA z-scores). Mann-Whitney *U-*tests were performed to determine group separation in terms of sympathetic activity (PEP *z*-scores). Statistical significance is marked by * for *p* < 0.05 and ** for *p* < 0.01. In regard to low scores of PA, men were identified by higher levels of parasympathetic activity than women. Men who scored low EC scores were characterized by higher parasympathetic activity than either men who had high EC scores or women who scored low scores of EC, while women who had high EC scores were identified by lower sympathetic activity than women who received lower EC scores.

To examine psychophysiological correlations for men and women separately, we created a four-dimensional comparison of data (see Figures 1, 2). To be consistent with previous literature regarding the two-space ANS model (Berntson et al., 2008; Brush et al., 2019), as well as for visualization purposes, we utilized *z*-scores of the physiological indices. Self-reported psychological measurements were split separately for each gender into high and low scores according to the median of each measurement. Normality of each physiological and psychological distribution was examined using a Shapiro-Wilk test. We next assessed whether each couple of distributions stem from separate populations. We probed for statistical significance by applying either one-sided *t*-test for data which follows normal distribution, or Mann-Whitney *U-*test for data which does not follow normal distribution.

## 3 Results

### 3.1 Physiological and psychological differences between the genders

Table 7 provides a summary of gender differences that were found within physiological and self-reported psychological parameters. Physiological indices were calculated as mean scores of the entire database. Considering physiological aspects, men are distinctly higher than women in measures of normalized RSA scores (men: mean 0.1812, std 1.0893, women: mean −0.0561, std 0.9657), CAB (men: mean 0.365, std 1.6763, women: mean −0.0695, std 1.3586) and CSAR (men: mean 0.3407, std 1.4468, women: mean −0.1729, std 1.3276). There was no significant difference between the genders in measures of normalized EDA scores (men: mean 0.1707, std 0.9825, women: mean −0.0602, std 1.0013), normalized PEP scores (men: mean −0.0797, std 1.2060, women: mean 0.0237, std 0.9313), CAR (men: mean 0.2055, std 1.5857, women: mean −0.0171, std 1.3664) and CSAB (men: mean 0.0209, std 1.4272, women: mean −0.0650, std 1.4380). Regarding psychological constructs, women reported themselves as significantly higher than men in parameters of state anxiety (men: mean 2.8274, std 0.7574, women: mean 3.2255, std 1.0421) and of EC (men: mean 3.9788, std 0.9154, women: mean 4.5621, std 0.9881), whereas no differences were found between the genders in PA (men: mean 3.2690, std 1.0996, women: mean 3.2862, std 1.1366) and NA (men: mean 1.7579, std 0.6818, women: mean 1.7457, std 0.6846).

**Table 7:** A summary of gender differences in psychological and physiological markers.

**Women dominance**	**Men dominance**	**No Gender difference**
EC^****^, state anxiety^*^	RSA^*^, CAB^*^, CSAR^**^	PA, NA, EDA, PEP, CAR, CSAB

Indices found to be higher in women are at the bottom left cell, the bottom middle cell portrays the indices for which men received higher scores, and psychological and physiological indicators for which no gender differences were found are at the bottom right cell. Physiological measures were calculated as mean values of the entire baseline period, for respiratory sinus arrhythmia (RSA) z scores (95 men, 307 women), electrodermal activity (EDA) *z*-scores (78 men, 221 women), pre-ejection period (PEP) *z*-scores (80 men, 269 women), cardiac autonomic balance (CAB, 80 men, 266 women) and regulation (CAR, 80 men, 266 women), cross-system autonomic balance (CSAB, 77 men, 217 women) and regulation (CSAR, 78 men, 217 women). Self-reported measures are reported for emotional contagion (EC, 95 men, 305 women), positive affect (PA, 96 men, 308 women), negative affect (NA, 96 men, 308 women) and state anxiety (38 men, 141 women). Welch's *t*-tests were conducted to determine whether men and women belong to separate populations, and the decisions are marked by ^*^ for confidence level of *p* < 0.05, ^**^ for *p* < 0.01 and ^****^ for *p* < 0.0001.

### 3.2 Psychophysiological relations

Spearman correlations were conducted between each pair of physiological and psychological indices (see Table 8). We found EC to be negatively correlated with the sympathetic EDA and PEP, as well as with CAR and CSAR. Both positive and negative affect dimensions were positively correlated with EDA and had negative links to CSAB. State anxiety had no significant correlations with any physiological measure. We then employed the Holm's method (Holm, 1979), designed to account for multiple hypothesis testing, to account for the relatively large number of correlations we conducted, and retested the significance of all Spearman's correlations. EC's relations to EDA and PEP as well as PA's correlation with EDA remained significant after this correction, while the other correlations did not.

**Table 8:** This table details all the correlations between the physiological parameters and psychological indices, for men (middle lines, blue) and for women (bottom lines, orange) separately and for the entire research population together (upper lines, black), and is calculated by Spearman's rank correlation.

**Indices**	**EC**	**PA**	**NA**	**State anxiety**
RSA	−0.05 (392)	−0.03 (396)	−0.00 (396)	−0.10 (173)
	−0.14 (92)	−0.04 (93)	−0.05 (93)	−0.15 (35)
	0.02 (246)	−0.02 (246)	−0.04 (246)	−0.15 (86)
EDA	−0.19^**#^ (294)	0.18^**#^ (295)	0.16^**^ (295)	−0.17 (108)
	−0.01 (76)	0.48^***##^ (76)	0.24^*^ (76)	−0.25 (32)
	−0.22^***#^ (216)	0.07 (215)	0.13 (215)	−0.11 (74)
PEP	−0.20^***##^ (340)	0.01 (343)	0.00 (343)	0.02 (139)
	−0.12 (77)	0.09 (78)	−0.00 (78)	−0.06 (25)
	−0.23^***##^ (218)	0.05 (217)	0.07 (217)	−0.03 (70)
CAB	0.10 (337)	−0.03 (340)	0.01 (340)	−0.08 (137)
	−0.09 (77)	−0.06 (78)	−0.06 (78)	−0.17 (25)
	0.18^**#^ (215)	−0.06 (214)	−0.06 (214)	−0.13 (68)
CAR	−0.13^*^ (337)	−0.01 (340)	0.05 (340)	−0.04 (137)
	−0.22 (77)	0.08 (78)	0.01 (78)	−0.21 (25)
	−0.07 (215)	0.02 (214)	0.05 (214)	−0.11 (68)
CSAB	0.11 (289)	−0.14^*^ (290)	−0.12^*^ (290)	−0.03 (105)
	−0.04 (75)	−0.38^***##^ (75)	−0.16 (75)	0.03 (31)
	0.17^*^ (212)	−0.08 (211)	−0.13 (211)	−0.06 (72)
CSAR	−0.14^*^ (290)	0.10 (291)	0.10 (291)	−0.18 (105)
	−0.05 (76)	0.25^*^ (76)	0.08 (76)	−0.25 (31)
	−0.13 (212)	0.02 (211)	0.06 (211)	−0.13 (72)

Degrees of freedom for each correlation calculation are indicated within the brackets, and represent the number of participants for whom correlations were calculated. Degrees of freedom for the entire research population may exceed the sums of degrees of freedom for men and women separately since not all participants reported on their gender. Statistical significance is marked by ^*^ for *p* < 0.05, ^**^ for *p* < 0.01 and ^***^ for *p* < 0.001. Due to the relatively large number of correlations calculated, we also employed Holm's correction method. Statistical significance after using Holm's correction method is indicated by ^#^ for *p* < 0.05 and ^##^ for *p* < 0.01.

Next, we split the data to men and women and recalculated all psychophysiological correlations. For men, PA is positively correlated with EDA and CSAR and negatively correlated with CSAB, while men's NA scores are positively correlated with their EDA measurements. After Holm's correction, the links PA have with EDA and CSAB remain significant. In women, however, only EC is significantly correlated with physiological activity, depicting positive links to CAB and CSAB and negative links to EDA and PEP. Most of those correlations remain significant after Holm's correction, and only the correlation between EC and CSAB does not.

In the next step, we split PA, NA, state anxiety and EC to high and low scores according to the median and compared the sympathetic and parasympathetic activities of groups of men and women who received high and low scores of each psychological aspect. A Shapiro-Wilk test was performed to check the normality of normalized RSA, EDA and PEP distributions. RSA *z*-scores of both men and women follow the normal distribution whereas EDA and PEP normalized scores of both genders do not. One-sided *t-*test was conducted for all RSA *z*-score distributions and revealed that men who received high EC marks (mean RSA: −0.056, std: 1.2) significantly differed from men who received low marks of EC (mean RSA:0.42, std: 0.95) in terms of their parasympathetic activities. This means that men who received low scores of EC also demonstrated significantly higher levels of parasympathetic activity during the baseline recording. Men who reported themselves as having low PA (mean RSA: 0.28, std: 1.1) and EC (mean RSA: 0.42, std: 0.95) had significantly higher RSA measurements than women who had low scores of PA (mean RSA: 0.002, std: 0.93) and EC (mean RSA: −0.07, std: 0.95). After applying the Holm's method for multiple hypothesis, however, only the separation between the genders in low EC scores remained significant. For all EDA and PEP distributions of both men and women Mann-Whitney *U-*tests were performed to determine whether there is a separation between individuals who received high or low scores in terms of sympathetic activity. Women's EDA recordings significantly differed between low (EDA median = −0.52, lower quartile = −0.91, upper quartile = 0.36) and high (EDA median = −0.17, lower quartile = −0.78, upper quartile = 0.664) PA and low (EDA median = −0.17, lower quartile = −0.77, upper quartile = 0.755) and high (EDA median = −0.54, lower quartile = −0.93, upper quartile = 0.219) EC scores, as did women's PEP recordings for scores of low (PEP median = 0.12, lower quartile = −0.29, upper quartile = 0.653) and high (PEP median = −0.018, lower quartile = −0.55, upper quartile = 0.385) EC and men's recordings of EDA for scores of low (EDA median = −0.38, lower quartile = −0.96, upper quartile = 0.3) and high (EDA median = 0.56, lower quartile = 0.093, upper quartile = 1.182) PA and low (EDA median = −0.13, lower quartile = −0.89, upper quartile = 0.466) and high (EDA median = 0.45, lower quartile = −0.44, upper quartile = 1.067) NA. All those separations remained significant after Holm's correction apart from women's EDA recordings for high and low PA marks. In other words, individuals who received low PA scores also demonstrated lower sympathetic activity as measured by EDA, men's sympathetic activity was higher in terms of EDA recordings for men who scored higher in NA, and women who had higher tendencies of EC demonstrated lower sympathetic activity, both in EDA and in PEP measures. In terms of separation between men and women, men's EDA measures were higher than women's who received high PA (men's EDA: median = 0.56, lower quartile = 0.093, upper quartile = 1.182; women's EDA: median = −0.17, lower quartile = −0.78, upper quartile = 0.664), NA (men's EDA: median = 0.45, lower quartile = −0.44, upper quartile = 1.067; women's EDA: median = −0.33, lower quartile = −0.78, upper quartile = 0.611) and EC (men's EDA: median = 0.19, lower quartile = −0.79, upper quartile = 0.941; women's EDA: median = −0.54, lower quartile = −0.93, upper quartile = 0.219) scores and in the groups who scored high anxiety marks (men's EDA: median = 1.0, lower quartile = 0.47, upper quartile = 1.186; women's EDA: median = 0.073, lower quartile = −0.65, upper quartile = 0.778). All of those separations remained significant after applying the Holm's method for multiple hypothesis. Figure 1 displays the mean normalized RSA and EDA scores of men and women within all experiments, while Figure 2 showcases mean normalizes RSA and PEP scores. Blue triangles and blue squares represent high and low scores (respectively) of men's PA (Figures 1A, 2A), NA (Figures 1B, 2B), state anxiety (Figures 1C, 2C), or EC (Figures 1D, 2D), while orange triangles and orange squares represent high and low scores (respectively) that women received in those psychological questionnaires. Error bars depict standard error.

### 3.3 Considering alternative models

**Figure 3:**
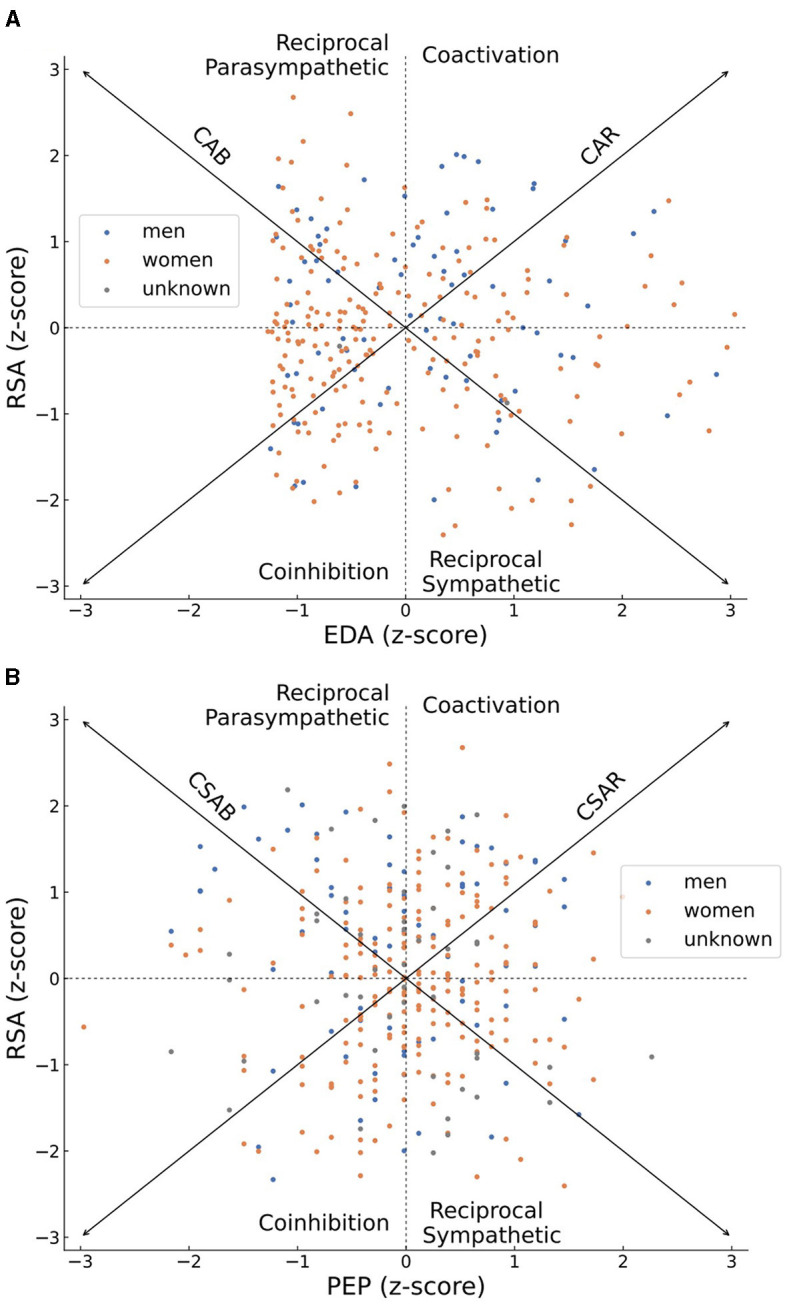
Scatter plots of the *z*-scores of individuals' parasympathetic (RSA) and sympathetic (EDA in **A** or PEP in **B**) activities, plotted on top of a bivariate representation of the autonomic space. Women's scores are colored in orange, men's scores are colored in blue, scores of individuals who did not report their gender are colored in gray. An individual who demonstrated an overall high parasympathetic activity during the recording period will be plotted on the high half of the graph, and an overall high sympathetic activity will place him or her at the right half of the graph. The overall distribution of sympathetic and parasympathetic activities of all participants is scattered and not concentrated across the CSAB or CSAR diagonal arrows, representing patterns of independent sympathetic and parasympathetic activities, and demonstrating a disagreement with either the bipolar model or the notion of total activation or disactivating of the autonomic nervous system.

The distributions of normalized RSA as well as normalized EDA scores or PEP scores across all subjects are plotted in Figures 3A, B, respectively. Women are shown by orange dots, men are plotted in blue while individuals who did not report their gender are depicted as gray dots. The distributions are plotted upon a bivariate representation of the autonomic space, primarily introduced by Berntson et al. (2008). The bivariate space depicts areas of coactivation of sympathetic and parasympathetic activity (CSAR in Figure 3A or CAR in Figure 3B), reciprocal parasympathetic activity (CSAB in Figure 3A or CAB in Figure 3B), reciprocal sympathetic activity and inhibition of both sympathetic and parasympathetic activity. The diagonal arrows of reciprocity (CSAB or CAB) represent a bipolar model, according to which an increase in sympathetic activity occurs on the expense of a decrease in parasympathetic activity and vice versa. The diagonal arrows of coactivation (CSAR or CAR) represent a mutual increase or decrease in the activity of both branches of autonomic nervous system. The general distribution of sympathetic and parasympathetic activities of participants in Figures 3A, B is not concentrated around either of those diagonal arrows, thus rejecting both the bipolar model and the notion of mutual activity or inhibition in favor of independency between the activities of the sympathetic and the parasympathetic branches of the ANS.

In order to conclude which physiological measures best predict each psychological aspect, as well as to determine if one-dimensional or two-dimensional physiological framework is more suitable for psychophysiological research, we created a set of linear regression models for each psychological index. We compared the amounts of explained variability produced by each model, as well as each model's Akaike information criterion (AIC) and Bayesian information criterion (BIC), both criterions for model selection which indicate a better fitting model the lower their scores are. Each psychological construct was modeled separately for men and for women, as well as for both genders together.

**Figure 4:**
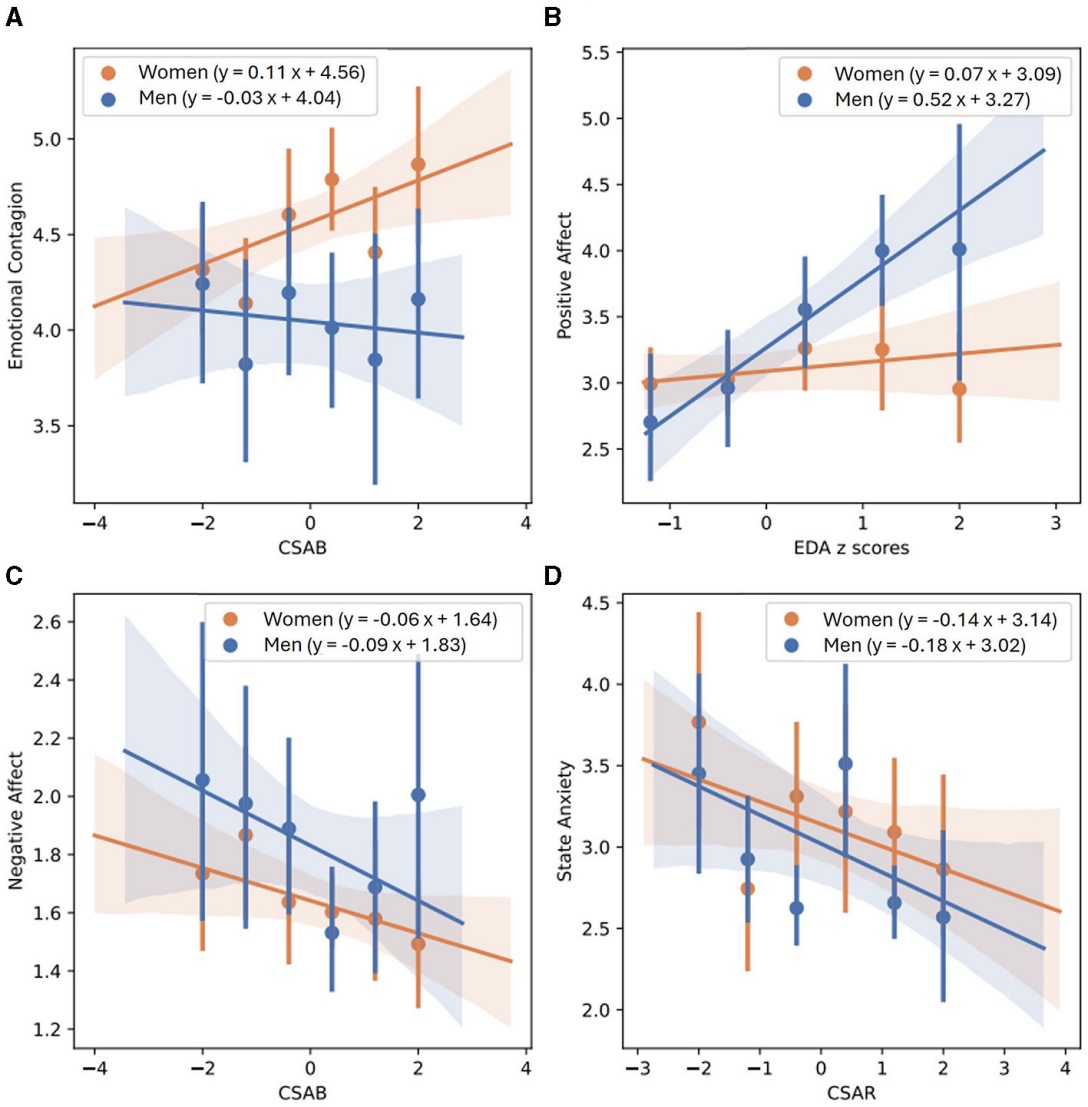
Linear regression between EC and CSAB **(A)**, PA and normalized EDA **(B)**, NA and CSAB **(C)** and state anxiety and CSAR **(D)**, separated for men and women. Men's best fit of linear regression is depicted by a solid blue line, mean values of each bin in represented by blue dots, and blue shaded areas demonstrate confidence intervals obtained using the bootstrap method. Women's linear regression best fit, mean values of separate bins and confidence intervals are depicted in orange.

Table 9 showcases linear regression models created to predict EC. When examining both genders together, the joint model of CSAB and CSAR explains the largest amount of variability (*R*-square = 0.0326) and produces the smallest values of AIC (828) and BIC (843), with only small differences from the model of EDA alone (*R*-square = 0.0300, AIC = 837, BIC = 848). This is also the case for the models created for women alone (joint model of CSAB + CSAR: *R*-square = 0.0398, AIC = 608, BIC = 621; EDA model: *R*-square = 0.0378, AIC = 615, BIC = 625). The comparison between models for men, however, yielded very different results, with CAB and CAR joint ability to predict EC far surpasses each of the other models (*R*-square = 0.0600). Linear regression fit of EC and CSAB for men and women are presented in Figure 4A.

**Table 9:** Linear regression models to predict emotional contagion (EC).

**Covariates**	***R*-square**	**AIC**	**BIC**
**Emotional contagion**			
**Both genders**			
RSA	0.00530	1,127	1,139
EDA	0.0300	837	848
PEP	0.0170	969	980
CAB + CAR	0.0214	963	978
**CSAB** **+** **CSAR**	**0.0326**	**828**	**843**
**Men**			
RSA	0.0271	253	261
EDA	9.11e-4	213	220
PEP	0.0114	220	227
**CAB** **+** **CAR**	**0.0600**	**218**	**227**
CSAB + CSAR	0.00961	212	221
**Women**			
RSA	7.86e-5	713	724
EDA	0.0378	615	625
PEP	0.0176	624	635
CAB + CAR	0.0192	620	633
**CSAB** **+** **CSAR**	**0.0398**	**608**	**621**

This table details the amount of explained variability (*R*-square), Akaike information criterion (AIC) and Bayesian information criterion (BIC) produced by each linear regression model to predict EC. The left column exhibits the physiological indices used as predictors in each model: respiratory sinus arrhythmia (RSA), electrodermal activity (EDA) and pre-ejection period (PEP) were entered as single covariates to separate models, whereas cardiac autonomic balance (CAB) and regulation (CAR) were assigned together as two predictors of the same model, as were cross-system autonomic balance (CSAB) and regulation (CSAR). The upper part of the table represents linear regression models calculated for both genders together, the middle part shows linear regression models for men and the lower part of the table depict linear regression models calculated for women. Based on the explained variability as well as the AIC and BIC scores, we chose the best fitting models to predict men's, women's, and both gender's EC/PA/NA/state anxiety scores, and highlighted them in bold letters.

Table 10 demonstrates all linear regression models calculated to predict PA. EDA predicts PA more accurately than any other model, with *R*-square = 0.0304 and only slightly higher scores of AIC and BIC than the combined model of CSAB and CSAR (EDA model: AIC = 889, BIC = 900; CSAB + CSAR model: *R*-square = 0.0289, AIC = 877, BIC = 891). The models for men repeat the same conclusions, with EDA predicting 22.5% of unexplained variability compared to 22.1% predicted by CSAB and CSAR together, and almost exactly the same AIC and BIC scores (EDA model: AIC = 218, BIC = 225; CSAB + CSAR model: AIC = 218, BIC = 227). For women, however, no physiological model accounts for even 1% of unexplained variability. A linear regression fit of PA and EDA for men and women is shown in Figure 4B.

**Table 10:** Linear regression models to predict positive affect (PA).

**Covariates**	***R*-square**	**AIC**	**BIC**
**Positive affect**			
**Both genders**			
RSA	4.13e-4	1230	1242
**EDA**	**0.0304**	**889**	**900**
PEP	2.17e-4	1,054	1,066
CAB + CAR	9.14e-4	1,049	1,065
CSAB + CSAR	0.0289	877	891
**Men**			
RSA	5.86e-5	293	301
**EDA**	**0.225**	**218**	**225**
PEP	0.00991	240	247
CAB + CAR	0.0134	242	251
CSAB + CSAR	0.221	218	227
**Women**			
RSA	3.55e-4	752	762
EDA	0.00350	658	668
PEP	0.00364	654	664
CAB + CAR	0.00378	649	663
CSAB + CSAR	0.00313	648	661

This table details the amount of explained variability (*R*-square), Akaike information criterion (AIC) and Bayesian information criterion (BIC) produced by each linear regression model to predict PA. The left column exhibits the physiological indices used as predictors in each model: respiratory sinus arrhythmia (RSA), electrodermal activity (EDA) and pre-ejection period (PEP) were entered as single covariates to separate models, whereas cardiac autonomic balance (CAB) and regulation (CAR) were assigned together as two predictors of the same model, as were cross-system autonomic balance (CSAB) and regulation (CSAR). The upper part of the table represents linear regression models calculated for both genders together, the middle part shows linear regression models for men and the lower part of the table depict linear regression models calculated for women. Based on the explained variability as well as the AIC and BIC scores, we chose the best fitting models to predict men's, women's, and both gender's EC/PA/NA/state anxiety scores, and highlighted them in bold letters.

NA predicting models are presented in Table 11. When calculated for both genders together, CSAB and CSAR measures account for 1.94% of all unexplained variability whereas EDA alone accounts for 1.35%. The AIC and BIC scores of both models are also similar (EDA model: AIC = 600, BIC = 611; CSAB + CSAR model: AIC = 591, BIC = 605). For men the difference between EDA model and CSAB and CSAR joint model is a little bigger, although measurements of model fit remain largely the same (EDA model: *R*-square = 0.0263, AIC = 169, BIC = 176; CSAB + CSAR model: *R*-square = 0.0387, AIC = 169, BIC = 178). For women, however, the joint model of CAB and CAR is the best predictor for NA, accounting for 2% of unexplained variability, whereas PEP and CSAB and CSAR models account for 1.29–1.52%. The CAB and CAR model is also preferable for women in terms of model fit (CAB + CAR model: AIC = 403, BIC = 416; PEP model: AIC = 410, BIC = 421; CSAB + CSAR model: AIC = 421, BIC = 434). The linear regression fit of NA and CSAB is presented in Figure 4C, separated for men and women.

**Table 11:** Linear regression models to predict negative affect (NA).

**Covariates**	***R*-square**	**AIC**	**BIC**
**Both genders**			
**Negative affect**			
RSA	3.22e-4	830	842
EDA	0.0135	600	611
PEP	0.00372	728	739
CAB + CAR	0.00393	723	738
**CSAB** **+** **CSAR**	**0.0194**	**591**	**605**
**Men**			
RSA	0.00683	202	210
EDA	0.0263	169	176
PEP	0.0101	175	182
CAB + CAR	0.0158	177	186
**CSAB** **+** **CSAR**	**0.0387**	**169**	**178**
**Women**			
RSA	0.00870	475	485
EDA	0.00758	429	439
PEP	0.0129	410	421
**CAB** **+** **CAR**	**0.0204**	**403**	**416**
CSAB + CSAR	0.0152	421	434

This table details the amount of explained variability (*R*-square), Akaike information criterion (AIC) and Bayesian information criterion (BIC) produced by each linear regression model to predict NA. The left column exhibits the physiological indices used as predictors in each model: respiratory sinus arrhythmia (RSA), electrodermal activity (EDA) and pre-ejection period (PEP) were entered as single covariates to separate models, whereas cardiac autonomic balance (CAB) and regulation (CAR) were assigned together as two predictors of the same model, as were cross-system autonomic balance (CSAB) and regulation (CSAR). The upper part of the table represents linear regression models calculated for both genders together, the middle part shows linear regression models for men and the lower part of the table depict linear regression models calculated for women. Based on the explained variability as well as the AIC and BIC scores, we chose the best fitting models to predict men's, women's, and both gender's EC/PA/NA/state anxiety scores, and highlighted them in bold letters.

State anxiety predicting models are presented in Table 12. CSAB and CSAR joint model is the best one to predict state anxiety for both genders together, accounting for 6% of unexplained variability and producing AIC of 292 and BIC of 303. EDA only accounts for 2.95% of unexplained variability while all other models explain < 1%. CSAB and CSAR remain the preferred model to use when separating the data for men and for women as well, accounting for 12.4% of unexplained variability for men and 3.31% for women, although in both cases the AIC and BIC scores are lower for PEP and for CAB and CAR models. Men's and women's linear fits for state anxiety and CSAR are shown in Figure 4D.

**Table 12:** Linear regression models to predict state anxiety.

**Covariates**	***R*-square**	**AIC**	**BIC**
**Both genders**			
**State anxiety**			
RSA	0.00822	494	504
EDA	0.0295	303	311
PEP	0.00232	411	419
CAB + CAR	0.00618	407	419
**CSAB** **+** **CSAR**	**0.0600**	**292**	**303**
**Men**			
RSA	0.0598	85.8	90.6
EDA	0.0559	80.7	85.3
PEP	0.00552	72.4	76.2
CAB + CAR	0.111	71.3	76.5
**CSAB** **+** **CSAR**	**0.124**	**76.5**	**82.4**
**Women**			
RSA	0.0169	250	258
EDA	0.0124	222	229
PEP	8.96e-5	206	213
CAB + CAR	0.00974	203	212
**CSAB** **+** **CSAR**	**0.0331**	**218**	**227**

This table details the amount of explained variability (R square), Akaike information criterion (AIC) and Bayesian information criterion (BIC) produced by each linear regression model to predict state anxiety. The left column exhibits the physiological indices used as predictors in each model: respiratory sinus arrhythmia (RSA), electrodermal activity (EDA) and pre-ejection period (PEP) were entered as single covariates to separate models, whereas cardiac autonomic balance (CAB) and regulation (CAR) were assigned together as two predictors of the same model, as were cross-system autonomic balance (CSAB) and regulation (CSAR). The upper part of the table represents linear regression models calculated for both genders together, the middle part shows linear regression models for men and the lower part of the table depict linear regression models calculated for women. Based on the explained variability as well as the AIC and BIC scores, we chose the best fitting models to predict men's, women's, and both gender's EC/PA/NA/state anxiety scores, and highlighted them in bold letters. Figure 4 demonstrates the linear regression of each psychological construct with one physiological index, separated to men (in blue) and women (in orange) regressions.

## 4 Discussion

This paper aimed to examine the superiority of two-dimensional models in explaining individual differences in psychological and physiological factors as well as psychophysiological associations, compared with separate SNS and PNS activity indicators. A secondary goal was to evaluate gender differences in the manifestation of those indices and psychophysiological relations. To this end, participants have completed questionnaires measuring their EC, PA, NA, and state anxiety. Physiological data were recorded during a social baseline period, and EDA, PEP, and RSA were calculated. Each psychological index was inspected both in the bipolar one-dimensional model of autonomic balance, comparing psychological indices to sympathetic activity (represented by EDA and PEP scores) and to parasympathetic activity (marked as RSA score), and in the framework of autonomic space model, comparing EC, PA, NA, and state anxiety to CAB, CAR, CSAB, and CSAR. Cardiac autonomic balance (CAB) and cross-system autonomic balance (CSAB) indicate the influence of the parasympathetic relative to the sympathetic branches of the ANS. Cardiac autonomic regulation (CAR) and cross-system autonomic regulation (CSAR) mark the overall ANS activation. Most psychological indices were correlated to some aspect of physiological activity, with pronounce differences between men and women in terms of psychophysiological correlations.

### 4.1 Psychophysiological relations

We examined EC as a trait related to physiological activity for the first time, and found negative correlations to sympathetic activity, measured by EDA and PEP scores, and to overall activation of the ANS, measured by CAR and CSAR scores. The literature links EC with social interaction (Hatfield et al., 1994; Butler, 2011), empathy, bonding, and attunement (Hatfield et al., 1994, 2011; Spoor and Kelly, 2004; Decety and Ickles, 2009; Neves et al., 2018). Sympathetic withdrawal is also connected to social engagement (Porges, 2001); therefore, EDA and PEP can be expected to correlate negatively with EC. The negative association between EC and CAR and CSAR indicates a decrease in total ANS activity in individuals with higher tendencies to catch others' emotions. Both high levels of total ANS activation (Berntson et al., 2008) and low levels of SNS activation (Schwartz et al., 1992; Airaksinen, 1999; Hohnloser, 2005; Billman, 2006a,b) are predictive of good health, and it will therefore be interesting to examine EC connection to health in the future.

As expected, PA was positively associated with sympathetic activity, measured by EDA scores, in line with previous studies (Neumann and Waldstein, 2001; Frazier et al., 2004; Shiota et al., 2011). Unlike our hypotheses, however, higher levels of PA were linked to lower scores of CSAB, meaning that high PA was characterized by SNS dominance, and not correlated with PNS activity. Those divergences from our original expectations, and indeed the inconsistency of physiological response to PA among different papers, may be attributed to the differences in questionnaires used to quantify PA in the literature. While we used the PANAS questionnaire, Demaree et al. (2006) used the Self-Assessment Manikin (Bradley and Lang, 1994), Frazier et al. (2004) adopted a combination of the Semantic Differential Scale (Mehrabian and Russell, 1974) and the Self-Assessment Manikin whereas Neumann and Waldstein (2001) applied an Affect Grid (Russell et al., 1989) and a 28-item questionnaire (Russell, 1980). The comparison of findings between different articles is complicated under such circumstances.

Keeping with the existing literature on NA (Demaree and Everhart, 2004; Oveis et al., 2009; Drachen et al., 2010), higher NA scores were associated with higher SNS activity, measured by EDA. Also consistent with our hypothesis, NA was negatively correlated with CSAB, indicating a sympathetic dominance in individuals with higher NA score. We predicted NA to have a negative association with RSA. However, no connection to parasympathetic activity was noted. A possible explanation for that gap may lay in the differences in autonomic patterns which emerge in response to distinct negative emotions, such as fear, disgust, and anger (for a review, see Shiota et al., 2011). Both NA (Watson and Pennebaker, 1989; Knapp et al., 1992; Billings et al., 2000; Kubzansky and Kawachi, 2000; Meeks and Murrell, 2001; Kiecolt-Glaser et al., 2002; Suls and Bunde, 2005; Powell et al., 2008; Finch et al., 2012; Pressman et al., 2013; Ambrona and López-Pérez, 2014) and high sympathetic activity (Schwartz et al., 1992; Airaksinen, 1999; Hohnloser, 2005; Billman, 2006a,b) are repeatedly associated with negative health consequences. The eliciting of high SNS response may be the cause through which NA influences health, and indeed some studies have demonstrated how health deficiencies associated with negative emotions are mediated by differences in cultural background (Consedine et al., 2002; Miyamoto et al., 2013; Pressman et al., 2013; Curhan et al., 2014) or in psychological processes (Bastian et al., 2012). Further research should be conducted to determine whether indeed health impairments caused by NA can be attributed to sympathetic activity.

No physiological index could predict state anxiety. Although there are other studies which reported no relation between state anxiety and physiological activity (Huwe et al., 1998; Spangler et al., 2002; Bajkó et al., 2012; Verkuil et al., 2014; Cho et al., 2019; Ringeisen et al., 2019; Bauerly and Jones, 2021), state anxiety is characterized, among other properties, by arousal of the ANS (Spielberger, 1966), and this lack of correlation with any physiological index is therefor still surprising. It may be explained by differences in the construction of our research compared with the common anxiety literature. Most studies tested physiological responses to a stressful situation, meant to trigger anxiety, compared with a neutral stimulus. Our physiological measurements, however, were obtained during a social baseline period designed to be devoid of any affective triggers. Under these low-anxiety circumstances, it stands to reason that there are no significant differences in physiological arousal levels among participants, and therefore correlations will be difficult to find.

### 4.2 Gender differences

Men and women demonstrated many differences in the distribution of distinct psychological and psychological indices, in psychophysiological relations and even in in terms of the physiological model which best describes each of their psychological variabilities.

We expected men and women to present no differences in any type of physiological activity. Indeed, we found both genders to express roughly the same levels of the sympathetic EDA, as was suggested by Boucsein (2012) and reported by Strohmaier et al. (2020), although contradicting other findings (Frazier et al., 2004; Codispoti et al., 2008; Bari, 2020). The sympathetic PEP was also found to be similar for both genders, a finding that is in line with the common research on the subject (Allen et al., 1993; Neumann and Waldstein, 2001; Brydon et al., 2012; Seddon et al., 2020). We found, however, that men had significantly higher levels of parasympathetic activity, a surprising result based on past research (Frazier et al., 2004; Codispoti et al., 2008; Cribbet et al., 2011; Moodithaya and Avadhany, 2012; Botek et al., 2018). However, there are works linking low resting RSA levels to higher levels of anxiety disorders (Friedman and Thayer, 1998; Friedman, 2007; Chalmers et al., 2014), which are in turn reported as higher in women (Angst and Dobler-Mikola, 1985; Weissman and Merikangas, 1986; Anderson et al., 1987; Líndal and Stefánsson, 1993; Kessler et al., 1994, 1995; Lewinsohn et al., 1998; Costello et al., 2003; Bruce et al., 2005; McLean et al., 2011), and in this regard it stands to reason that women will express lower levels of baseline RSA. Women also demonstrated lower parasympathetic dominance (expressed by CAB), previously associated with greater health risk factors (Brush et al., 2019; Alen et al., 2020) as well as with higher levels of depressive symptoms (Stone et al., 2020). Time and again, women are reported to be about twice as likely as men to develop depression (for reviews see: Piccinelli and Wilkinson, 2000; Nolen-Hoeksema, 2001; Girgus and Yang, 2015). Further research is required to determine whether differences in physiological activity are associated to this elevated risk for women.

EC scores as well as EC's psychophysiological correlations differed for men and women. As expected, men exhibit lower EC scores than women, in line with previous research on the subject (Hall, 1978; Buck, 1984; Doherty et al., 1995; Surakka and Hietanen, 1998; Wild et al., 2001; Sonnby-Borgström et al., 2008; Magen and Konasewich, 2011; Manera et al., 2013; Fairbairn et al., 2015). Interestingly, there were also differences in psychophysiological correlations between the genders. While men EC scores exhibited no relations to any physiological activity, women reported higher levels of EC in accordance with lower sympathetic activity, measured by EDA and PEP, and with higher parasympathetic dominance, manifested by higher scores of CAB and CSAB. Those findings are partially in line with a recent study (Tracy and Giummarra, 2017) which examined gender differences in empathy to pain and its relation to parasympathetic activity, and found no such relations in men, and a negative correlation between baseline parasympathetic activity at rest and women's empathy trait. Our finding of women's association between EC and parasympathetic dominance, as well as our finding of higher parasympathetic activity in the group of men individuals who received higher scores of RSA, is in agreement with Stellar et al. (2015), who reported a positive relation between parasympathetic activity and compassionate tendencies. Notably, 72% of their participants were women. It is interesting to note that in our study, neither gender demonstrated any relation between EC and overall ANS activation, although such a connection was evident when taking both genders together into account.

In regard to affect, both genders reported similar levels of PA and NA. Although most past studies reported women to have higher PA and NA scores (Fujita et al., 1991; Grossman and Wood, 1993; Thomsen et al., 2005; Zuckerman et al., 2017; Batz and Tay, 2018), some reported no such differences (Neumann and Waldstein, 2001; Cribbet et al., 2011; Volante et al., 2016). We did find, however, differences in psychophysiological correlations between men and women. Women's PA and NA scores demonstrated no significant correlations with any physiological measure, similarly to several studies (Levenson et al., 1994; Demaree et al., 2006). Women who belonged to the group of higher PA scores, however, were characterized by higher EDA recordings, supporting other works which reported a positive correlation between PA and parasympathetic activity in groups consisting mostly of women (Oveis et al., 2009; Kop et al., 2011; Wang et al., 2013). Men reported higher levels of PA and of NA in accordance with higher sympathetic activity, measured by EDA. Previous studies have similarly showed a positive correlation between NA and sympathetic activity in men (Levenson et al., 1994) or for both men and women (Anderson et al., 2017), while Neumann and Waldstein (2001) demonstrated a link between PA and sympathetic activity for both genders. Men's levels of PA were also correlated with overall higher activation of the ANS, measured by CSAR, and with sympathetic dominance, indicated by lower levels of CSAB. While as one united group men and women's NA scores were related to sympathetic dominance, this relation cannot be found separately in any of the two groups. This separation to men and women also enabled our conclusion that all PA correlations with physiological activity measured in the general sample of both genders actually stemmed solely from the men's data.

As for state anxiety, women's levels of anxiety were significantly higher than men's levels, in line with many studies (Angst and Dobler-Mikola, 1985; Weissman and Merikangas, 1986; Anderson et al., 1987; Bourdon et al., 1988; Líndal and Stefánsson, 1993; Campbell and Rapee, 1994; Kessler et al., 1994, 1995; Lewinsohn et al., 1998; Poulton et al., 2001; Muris and Ollendick, 2002; Costello et al., 2003; Bruce et al., 2005; McLean et al., 2011), but contradicting others (Carrillo et al., 2001; Brand and Schoonheim-Klein, 2009; Limbu et al., 2010; Strohmaier et al., 2020). As for correlations between state anxiety and physiological activity, both genders demonstrated none. There are apparent gender differences in the physiological response to stress (for a review, see Donner and Lowry, 2013), and Kong et al. (2022) even showed that men's patterns of physiological activity can discriminate between high and low trait anxiety individuals much more reliably than women's patterns. Therefore, our results in this regard are unexpected.

### 4.3 Autonomic space model

The main goal of this study was to investigate the autonomic space models as frameworks for physiological correlations with psychological measures and to inspect their superiority over the one-dimensional ANS models. In our study, we chose to employ both the cardiac autonomic balance (CAB) and regulation (CAR) model and the refined framework of cross-system autonomic balance (CSAB) and regulation (CSAR). Since the CSAB-CSAR framework is based on EDA, a sympathetic index of the eccrine system (Fowles, 1986; Shields et al., 1987; Dawson et al., 2007), and RSA, a parasympathetic index of the cardiac system (Cacioppo et al., 1994; Malik et al., 1996; Berntson et al., 1997), whereas the CAB-CAR model represents purely cardiac activity (Sherwood et al., 1990; Berntson et al., 1994a; Cacioppo et al., 1994; Benevides and Lane, 2013; Stone et al., 2020), the usage of both frameworks allowed us to examine physiological correlates of psychological traits and processes in a comprehensive ANS context.

The distribution of sympathetic and parasympathetic scores in the two-dimensional autonomic space (Figure 3) suggests a clear preference for the autonomic space models. The classic one-dimensional model requires a decrease in parasympathetic activity to accompany an increase in sympathetic activity and vice versa (a slope of −1 in the two-dimensional space, concentrating around the CAB or CSAB diagonal arrows). This relationship does not manifest in our results, nor does a repeated co-activation or co-inhibition of the two ANS branches (a slope of +1 in the two-dimensional space, concentrating around the CAR or CSAR diagonal arrows). Our results reinforce that the PNS and the SNS can be activated independently from one another (Berntson et al., 1991; Berntson and Cacioppo, 2007; Stone et al., 2020). We will note that this clear support of the autonomic space model is only provided here for a baseline period, in which no stimulation was given to the participants. Further research is needed to determine whether this is the preferred physiological framework in cases of activity, interaction, or stimulation.

To further investigate which physiological model best predicts each psychological aspect, we conducted a series of linear regression calculations. When considering both genders together, we found that the CSAB-CSAR model best predicted the variability of EC, NA and state anxiety, whereas EDA fitted PA better. CSAB-CSAR model was the best fitting for most psychological constructs calculated for men and women separated as well, while only men's PA variability was better predicted by the one-dimensional EDA. We therefore conclude that at rest, two-dimensional models, and specifically the CSAB-CSAR model, are the most predictive of most psychological aspects.

### 4.4 Limitations and strengths

Our study has several limitations. The first is the employment of self-reported questionnaires, subjecting the psychological indices to bias (Wang, 2015). The second limitation concerns the structural design of the research. The focus of our study was correlation measurements, rendering it impossible to conclude any reasoning or direction of effect between psychology and physiology. The third limitation regards the measurement context. Our physiological measurements were obtained during a baseline period, during which an individual is supposedly awake, at rest, and removed from any stimuli. However, the level of alertness a subject is experiencing during a baseline period is unclear, and there are possible stress-inducing stimuli that may affect them, such as electrode hookups and the company of other individuals. See Fishel et al. (2007) for a broader consideration of the baseline procedure. The fourth and final limitation concerns the research population. As in many psychophysiological studies, most of our subjects were women. A careful consideration of all results obtained for men is warranted, as well as the comparisons between the genders, due to this imbalance. A properly balanced research sample will be needed to strengthen and verify the associations we found here in future research.

Two strengths of this research are the relatively large number of subjects, thanks to which we can draw more robust conclusions (Machin et al., 1987; Whitley and Ball, 2002), and the usage of several physiological frameworks, allowing for a comprehensive understanding of the relationship between the psychological measures and the physiological activity. This paper was one of the first to examine the psychological aspects of the autonomic space model and expanded our understanding of the psychology-physiology link.

## Data availability statement

The data analyzed in this study is subject to the following licenses/restrictions: deidentified data will be made available upon request from the corresponding author. Requests to access these datasets should be directed to ilanit.gordon@biu.ac.il.

## Ethics statement

The studies involving humans were approved by Department of Psychology IRB Bar-Ilan University. The studies were conducted in accordance with the local legislation and institutional requirements. The participants provided their written informed consent to participate in this study.

## Author contributions

YMS: Formal analysis, Investigation, Methodology, Visualization, Writing – review & editing. YXJM: Formal analysis, Investigation, Validation, Visualization, Writing – original draft, Writing – review & editing. MAD: Writing – original draft. SG: Methodology, Supervision, Writing – review & editing. RPB: Methodology, Supervision, Writing – review & editing. IG: Conceptualization, Funding acquisition, Methodology, Resources, Supervision, Writing – review & editing.
